# Crystal structure of the fungal mannosyltransferase Och1 reveals active site primed for *N*-glycan binding

**DOI:** 10.1371/journal.pone.0329259

**Published:** 2025-07-31

**Authors:** Emma T. R. Kelly, Dmitry Rodionov, Barry Sleno, Pedro A. Romero, Albert M. Berghuis

**Affiliations:** 1 Department of Biochemistry, McGill University, Montréal, Québec, Canada; 2 Centre de recherche en biologie structurale, McGill University, Montréal, Québec, Canada; 3 McGill Antimicrobial Resistance Center, Montréal, Québec, Canada; 4 Department of Microbiology and Immunology, McGill University, Montréal, Québec, Canada; Tokyo University of Pharmacy and Life Sciences: Tokyo Yakka Daigaku, JAPAN

## Abstract

The outermost layer of a fungi’s cell wall serves as the organism’s point of first contact with its environment, or host. Heavily glycosylated glycoproteins anchor a complex meshwork of branching mannose chains, forming the outer cell wall layer in most yeast and mold species. Outer mannan chains are composed of large polymannose branching glycans attached to the universal eukaryotic *N*-glycan GlcNAc_2_Man_8_ core. Synthesized in the endoplasmic reticulum, the core *N*-glycan is transferred to the Golgi apparatus, where the first fungi-specific reaction takes place. In the *cis*-Golgi, Och1 (Outer chain elongation 1) plays a central role in initiating outer mannan cell wall synthesis by transferring a single α-1,6-mannose residue to the *N*-GlcNAc_2_Man_8_ core. Playing a vital role in fungal biology, fungal cell wall synthesis proteins have long since been thought as attractive options in the search for a fungi-specific drug target. *Saccharomyces cerevisiae* Δ52-Och1 was expressed in *Pichia pastoris*. Here, the first X-ray crystal structure of a fungal Och1 protein is reported, determined to 2.0 Å. Molecular modeling of ligand binding and sequence analysis has revealed a highly conserved substrate binding site, rationalizing Och1 target specificity for the *N*-GlcNAc_2_Man_8_ glycan.

## Introduction

Situated exterior to the cell membrane, the fungal cell wall has become an increasingly attractive option in the search for a fungi-specific therapeutic target, particularly against yeast *Candida* and filamentous *Aspergillus* fungi species [1–3]. Representing ~40% of the fungal cell volume, the cell wall is the fungi’s point of first contact with its host’s membrane [4,5]. The wall’s outermost layer is primarily composed of mannoproteins. These proteins are extensively *N*-glycosylated with large branching polymannose chains (100–300 mannose residues per *N*-glycan) [4,5]. This forms a meshwork of mannan which can modulate the host’s immune response and mask the fungi’s initial presence [3,6–9]. Moreover, when mannosylation has been genetically prevented, fungal cells exhibit reduced cell growth and diminished virulence [10–12]. As one of the fungi’s major virulence factors, cell wall biosynthesis and the enzymes responsible for mannan chain elongation are of key interest with regards to understanding fungal biology [1,7,8]

**Fig 1:**
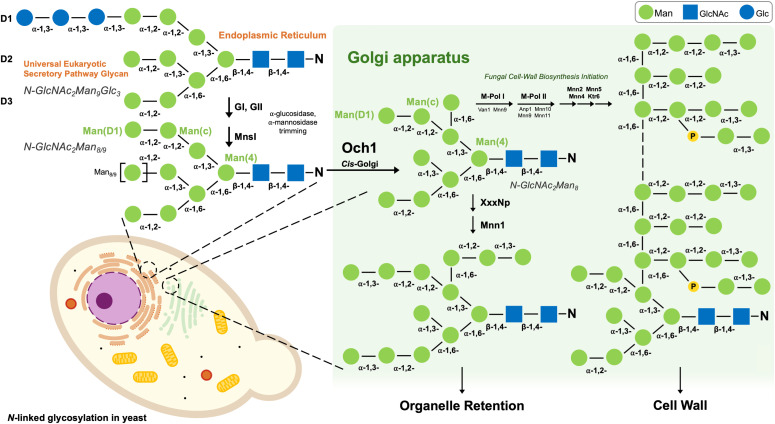
Outer cell wall mannan synthesis pathway in *S. cerevisiae*, taking place in the yest Golgi apparatus. Universal eukaryotic *N*-linked glycan, GlcNAc_2_Man_9_Glc_3_, is trimmed down to *N*-GlcNAc_2_Man_8/9_ prior to transfer to the cis-Golgi, where mannan synthesis begins with the first addition of α-1,6-Man by Och1. Complex mannan glycans are synthesized downstream by α-1,6-mannosyltransferase complex M-Pol I and II. Mannan branching is then formed by subsequent α-1,2- and α-1,3-mannosyltransferases, before deposition on the cell wall surface. Schematic proportions are not to scale.

As part of the secretory pathway, eukaryotic *N*-linked glycosylation starts in the endoplasmic reticulum (ER) where the universal eukaryotic glycan GlcNAc_2_Man_9_Glc_3_ is transferred to the N-X-S/T sequon of an accepting glycoprotein [2,4]. This glycan’s most terminal residues then undergo glucosidase and mannosidase trimming of as part of the eukaryotic calreticulin (CRT)/calnexin (CNX) protein folding quality control cycle, yielding the universal *N*-GlcNAc_2_Man_8_ glycan, before transportation into the *cis*-Golgi apparatus (Fig 1) [4,13,14]. *N*-GlcNAc_2_Man_8_ is a high-mannose glycan with three modifiable branches (given the nomenclature: D1, D2, and D3 branches) (Fig 1). Upon entry to the Golgi for maturation and cellular sorting, the *N*-glycan’s fate is decided by several glycosyltransferases (GTs) and/or glyco-active enzymes [2,4,13]. It is within the Golgi where the yeast and mammalian eukaryotic secretory pathways diverge [13]. In yeast and other fungi, *N-*GlcNAc_2_Man_8_ is modified upon entry to the *cis*-Golgi by the CAZy GT-32 transmembrane α-1,6-mannosyltransferase (MT) Och1 (Outer chain elongation 1) [4,13,15]. Och1 acts as the first fungi-specific step by which *N-*GlcNAc_2_Man_8_ is dedicated to higher-mannose modification [2,4,13]. Och1 transfers a single α-1,6-linked mannose onto an internally (α1−2)Man(α1−3) linked residue on the D1-mannosyl branch (between the Man(C) and innermost Man(4) residues) and from there, the glycan is either marked for the outer cell wall or organelle retention (remaining as a “core-type” glycan) (Fig 1) [5,13,15,16]. The α-1,6-Man residue may be further extended by α-1,2- and α-1,3-Man residues for organelle retention (thought to be done by an unknown α-1,2-MT and GT-71 α-1,3-MT Mnn1, respectively) (Fig 1) [17]. Outer cell wall synthesis, alternatively, begins following Och1 activity with continued α-1,6-Man extension (Fig 1) [5,18,19]. The heterodimeric complex M-Pol I (Van1 and Mnn9) is responsible for initiating extension with the addition of approximately ten α-1,6-Man residues [5,19]. The mannose backbone is then further extended by an additional ~50 α-1,6-Man residues by the putative heterotetrameric complex M-Pol II (Anp1, Mnn9, Mnn10, and Mnn11) (S1 Table) [5,19,20]. The α-1,6-mannan backbone is further mannosylated, in the *medial-*Golgi, forming α-1,2-, α-1,3-Man, and phosphomoannose branches (Fig 1) [5,18,21]. Finally, the mannoprotein exits the *trans*-Golgi via vesicular transport to be deposited at the cell membrane and released into the periplasmic space [5,13]. Mannoproteins are anchored into the inner wall’s β-glucan component to form the fungal cell wall outer layer mannan meshwork [6].

Och1, a family 32 glycosyltransferase, is a predicted retaining GT (ret-GT); identifiable by a hydrophobic (or aliphatic) residue at the catalytic DxD motif x-position [22–25]. Despite numerous studies on GTs that catalyzed a reaction in which the stereochemistry around the anomeric C1 carbon is retained, a general consensus regarding the enzyme mechanism used for this does not exist [25–27].

**Fig 2:**
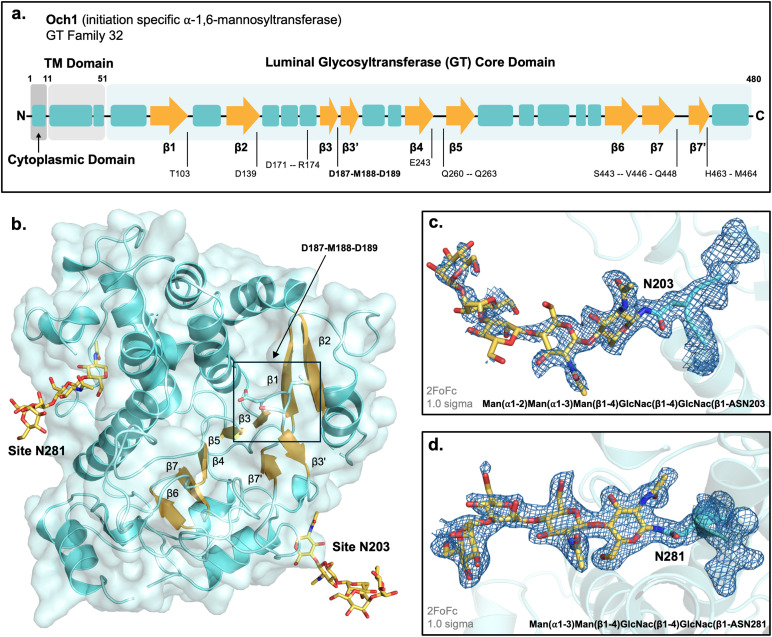
X-ray crystal structure of apo *S. cerevisiae* Och1. **a.** Secondary structural elements of Och1, shown by schematic (α-helices shown in blue, β-strands shown as yellow arrows). **b.** X-ray crystal structure of apo *S. cerevisiae* Δ52-Och1 with eukaryotic glycosylation at positions N203 and N281 (PDB: 9N3S). DxD motif at position D187-M188-D189 between β3- and β3’-strands indicated in black box. **c.** Glycosylation of positions N203 and **d.** N281 showing 2F_o_-F_c_ electron density, 1.0 sigma.

Functional studies have explored the vitality of cell wall MTs, establishing their necessity for cell growth, stability, and virulence [10–12]. Nevertheless, the proteins belonging to this cell wall synthesis pathway remain structurally uncharacterized. To date, Mnn9, a monomeric GT-62 protein subunit of M-Pol I and II, remains the only other published structure (PDB: 3ZF8) [19]. Persisting to today, experimental structural study remains non-trivial with many GTs requiring disulfide bonding, eukaryotic chaperones, and varying *N-*linked glycosylation profiles [26,27]. This is further complicated by their fast substrate hydrolysis, yielding structures lacking whole substrate bound states. Here, we present the first X-ray crystal structure of a fungal Och1 protein. *Saccharomyces cerevisiae* Δ52-Och1 was solved with 2.0 Å resolution, showing protein *N-*linked glycosylation patterning, with a final R/R_free_ of 0.1978/0.2428 (Fig 2). *In silico* modeling has been performed to assess both potential substrate- and product-bound states. This is the third published CAZy GT32 structure [23–25].

## Materials and methods

### Materials

TOPO Cloning and *Pichia pastoris* Expression kits were obtained from Invitrogen. Macro-Prep (CHT) Ceramic Hydroxyapatite supports (type 1 20 µM) were obtained from Bio-Rad (Mississauga, Ont., Canada). DEAE-Tricacryl® M was from Sigma-Aldrich Canada Ltd. (Oakville, Ont., Canada). CM Sepharose FF and Sephadex G-15 from GE Healthcare Canada (Mississauga, ON, Canada). Oligonucleotides were synthesized by Biocorp Inc. (Montréal, Qué., Canada). All other chemicals were reagent grade. All protein structures were assessed in PyMOL [28].

### Plasmid construction

The DNA sequence encoding for the Δ52-Och1 soluble GT-core domain (i.e., excluding the *N*-terminal cytoplasmic and transmembrane domains) was amplified from *S. cerevisiae* genomic DNA and subcloned into pCR2.1-TOPO vector, according to TOPO kit manufacturer’s instructions. The subsequent insert containing Δ52-*OCH1* was excised by restriction enzymes KpnI/XbaI and subcloned into pPICZαA vector as directed by the manufacturer.

### Cloning and recombinant protein expression of *S. cerevisiae* Och1 in *P. pastoris*

Δ52-**Sc*OCH1* containing pPICZαA vector was linearized with BstXI restriction enzyme and transformed into *Pichia pastoris* strain X-33 competent cells by electroporation. Zeocin-resistant transformants were selected and plasmid uptake was confirmed by PCR via 5’AOX1 and 3’AOX1 sites. 500 mL of BMGY medium (1% w/v yeast extract, 2% w/v peptone, 1.34% yeast nitrogen base, 4x10^-5^% biotin, 1% v/v glycerol, 100 mM TRIS-HCl, pH 7.0) was inoculated with 0.5 mL of an overnight preculture. Yeast cultures (500 mL) were grown for 48 hr at 30 ºC in a shaker incubator. The cells from individual flasks were subsequently aseptically pelleted by centrifugation and resuspended in 250 mL BMMY induction medium (replacing BMGY glycerol containing media with 0.5% v/v methanol). Expression was carried out for 24 hr at 30 ºC. This process was repeated three times, and each time the pellets were resuspended in fresh BMMY. Final media was clarified by centrifugation and stored at −85 ºC.

### Purification of *S. cerevisiae* Och1 from *P. pastoris*

Δ52-*Sc*Och1 containing medium was thawed at 4 ºC, pooled and clarified by centrifugation, and filtered (0.22 μM membrane). Pooled medium was concentrated using Pellicon®2 unit equipped with 30 kDa MWCO cassette (Millipore). Concentrated medium was filter-dialysed against 1 mM NaCl pH 7.0 using the same concentrator. Och1 was captured by passing the medium through Macro-Prep (CHT) ceramic hydroxyapatite column (equilibrated in 1 mM NaCl), and eluted in a single step with 100 mM KF buffer. Och1 containing fractions were pooled and applied to DEAE-trisacryl ion-exchange column (50 mM Bis-TRIS pH 7.0) and eluted with 0–250 mM NaCl gradient in the same buffer. Nearly pure Och1 fractions were then loaded onto a CM (carboxymethyl) Sepharose cation exchange column (equilibrated in 50 mM AcONa pH 4.5), and eluted with a 0–500 mM NaCl gradient. Purified Och1 at 15 mg/mL was stored in 10 mM Bis-TRIS pH 6.5 at 4 ºC. All buffer exchange steps were carried out by desalting on a Sephadex G-15 column. The protein was followed throughout the purification by SDS-PAGE. The final yield of Och1 was approximately 1 mg per litre of expression media.

### Crystallization and data collection

Crystallization was a non-trivial process due to huge variation in Och1 protein glycosylation profile, hindered by glycan-heterogeneity in size and number of sites occupied. *Sc*Och1 crystals were grown in MRC2 plates, at 22°C, by sitting-drop vapour diffusion. Diffraction-quality crystals were obtained from 2 µL drops, containing equal parts protein (15 mg/mL Δ52-*Sc*Och1 in 10 mM Bis-TRIS pH 6.5) and mother liquor (0.1 Bis-TRIS pH 6.5, 25% PEG 3350, 30 mM NaF). Resulting crystals were cryo-protected by flash-swiping through the mother liquor and supplemented with 50% PEG 3350. Data collection was performed on a Rigaku MicroMax-007 HF copper source equipped with VariMax-HF optic, and Saturn 944 + camera on an AFC11 (wavelength 1.54 Å).

### Structural determination and refinement

X-ray crystal data was processed using the HKL2000 software suite [29]. The crystal structure of Och1 was solved by molecular replacement (MR) using the PHENIX software package [30]. An AlphaFold2 predicted model of *S. cerevisiae* Och1 was used as the search model (S1 and S2 Figs) [31,32]. Molecular replacement rotation and translations were obtained for two Och1 copies within the asymmetric unit. Model refinement was carried out in successive rounds of real- and reciprocal-space refinement using PHENIX and COOT [30,33]. Model modifications, corrections, and additions were decided based on 2F_o_-F_c_ and F_o_-F_c_ electron density maps. Model refinement was performed until no significant improvements could be achieved, judged by R/R_free_ value decrease (Table 1).

**Table 1:** X-ray crystal structure of apo *S. cerevisiae* Och1.

Data Collection		
Wavelength		1.54
Resolution range (Å)		24.46–2.01 (2.06–2.01)
Space Group		P1
Unit cell	a, b, c (Å)	43.5, 57.1, 84.1
	α, β, λ (°)	85.6, 75.3, 75.1
Total reflections		175381 (7965)
Unique reflections		44905 (2104)
Completeness (%)		89.78 (58.20)
Mean I/sigma(I)		4.81 (4.34)
Wilson B-factor (Å^2^)		24.78
R_*merge*_		0.2931 (0.303)
CC1/2		0.843 (0.809)
CC*		0.957 (0.946)
Multiplicity		3.8 (3.3)
**Refinement Statistics**		
No. of reflections		44905
No. used for R-free		1999
R_*work*_/R_*free*_		0.1978/0.2428
Average B-factor (Å^2^)		30.5
Clashscore		6.1
No. of atoms	Non-hydrogen	6190
	Macromolecule	5812
	Ligand	153
	Solvent	225
Ramachandran (%)	Favoured	97.6
	Allowed	2.1
	Outliers	0.3
	Rotamer outliers	2.0

### Evaluation of functional and structural conservation

NCBI BLAST (https://blast.ncbi.nlm.nih.gov/Blast.cgi) and Clustal Omega (https://www.ebi.ac.uk/jdispatcher/msa/clustalo) were both used to perform sequence alignment of twenty-two Och1 homologs (encompassing both yeast and mold species) [34–36]. Och1 homolog sequences were retrieved from the NIH Protein database (https://www.ncbi.nlm.nih.gov/protein/) [37]. Multiple sequence alignment assessment was carried out by AL2CO scoring [38]. Och1 structural homology was assessed by performing a Dali Protein Structure Comparison Server full PDB search (http://ekhidna2.biocenter.helsinki.fi/dali/) [39].

### *In silico* substrate-complex prediction and higher mannan glycan molecular modeling

Modeling of the ternary complex of Och1 in both the substrate-bound and product-bound states were performed in stages. Initial modeling of the GDP moiety was performed using AlphaFold3 (https://alphafoldserver.com/about) [40]. This yielded a satisfactory approximate prediction of GDP and Mn^2+^ placement which was consistent with known cation-coordinated nucleotide binding within a GT-A fold. For modeling of the substrate-bound state, the nucleotide-sugar donor, GDP-Man, and glycan acceptor substrates were built by expanding on the AlphaFold3 placed GDP molecule with Molecular Operating Environment (MOE) (S3 Fig) [40,41]. Ligand modeling of the GDP-Man donor and manno-glycan acceptor (the *N-*GlcNA_2_Man_8_ glycan D1 branch) placement were improved with successive iterations of energy minimization and conformational searches using MOE. The decision to model the acceptor glycan D1 branch was made based on its agreement with Och1 pocket size and proximity. Improvements to ligand placement were made such that results were consistent with known GT-A binding site theory [25,26,42]. Modeling of the product-bound state followed the same strategy, using the substrate-bound state as a starting point (S3 Fig).

## Results

### Overall structure of Och1 follows a sequence predicted GT-A folding pattern

The crystal structure of Δ52-*Sc*Och1 was solved, in the apo state, at a resolution of 2.0 Å, R/R_free_: 0.1978/0.2428 (Fig 2, Table 1). Co-crystallization in the presence of GDP-Man nucleotide donor substrate did not yield electron density indicative of ligand binding. The N-terminal and transmembrane helix domains, accounting for the first 51 residues of the primary sequence, were excluded from the construct used. The Och1 crystal data reveals electron density covering the C-terminal GT-core domain of the type II transmembrane protein, with the exception of some flexible loop regions and the C-terminal tail, which could not be resolved due to poor electron density (missing A-chain residues: K72 – Q76, H315 – E327, N374 – T400, F465 – K480) (shown in Fig 2). Noteworthy, regions of poor electron density correspond to segments that were modeled with low confidence by AlphaFold (S1 Fig) [31,32,39].

The three-dimensional structure of Och1 confirms the GT-A folding structural elements as predicted by its sequence, adopting the canonical GT-A single Rossmann-like folding pattern (Fig 2b) [25,26]. Och1 exhibits the archetypical β-sheet core, flanked by several α-helices, with an additional pair of smaller anti-parallel β-strands housing the glycosyltransferase catalytic DxD motif, at position D187-M188-D189 (Fig 2b). Furthermore, there are four sequence predicted *N*-glycosylation sites at N203-K204-S205, N281-I282-T283, N341-W242-T343, and N393-D394-T395 (glycosidase treatment was not applied to the sample). Unsuccessful bacterial expression attempts revealed *N*-glycosylation is needed for Och1 expression, thereby necessitating eukaryotic expression in the case of *Sc*Och1. While needed for ScOch1 protein expression, N203, N281, and N341 glycosylation sites are not conserved across all fungal Och1 homologs. For positions N203 and N281, the electron density was such that partial glycosylation could be resolved. Our model contains Man(⍺1–2)Man(⍺1–3)Man(β1–4)GlcNAc(β1–4)GlcNAc(β1-N203 and Man(⍺1–3)Man(β1–4)GlcNAc(β1–4)GlcNAc(β1-N281, patterning consistent with eukaryotic secretory pathway protein glycosylation (Fig 2c and 2d) [13,14]. Unfortunately, poor electron density at both N341 or N393 rendered us unable us to model any posttranslational glycosylation modifications for these two sites.

### Och1 presents an evolutionarily conserved nucleotide-sugar binding pocket

**Fig 3:**
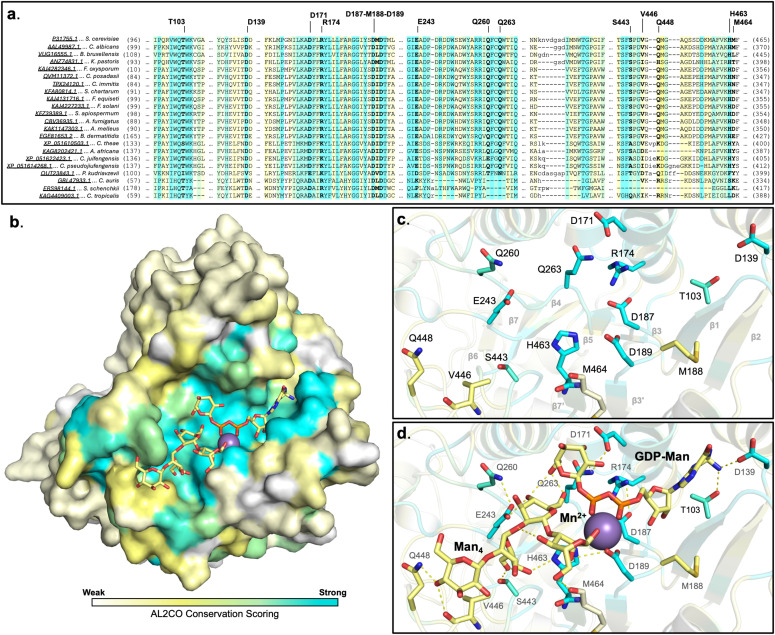
Mapped sequence conservation across Och1 yeast and mold homologs. Conservation of residues, scored via AL2CO, ranging from least to most is coloured on a spectrum of white (least) to yellow to cyan blue (most). **a.** Multiple sequence alignment of residues belonging to the Och1 active site. **b.** Global fold sequence conservation, showing modeled GDP-Man and Man_4_ glycan binding in the substrate-bound state. **c.** Pocket residues identified as potential ligand binding facilitators, compared to **d.** modeled substrate state bound to GDP-Man and Man_4_ glycan.

Amino acid sequence conservation across twenty-two Och1 yeast and mold species homologs is represented in Fig 3. Sequence conservation, as seen by colour mapping onto the structure, is largely localized to the Och1 active site (i.e., the nucleotide-sugar donor substrate and acceptor binding pocket) (Fig 3). This thereby implies the functional importance of that region to Och1 mannosylation activity.

Sequence conservation of the active site residues appear to be consistent with known elements common to the core domain of a GT-A folded glycosyltransferase (Fig 3c). Of interest, conserved residues T103, D139, D171, R174, Q263, and H463, in addition to a conserved DxD motif (D187-M188-D189), are located such that each have the potential to play a role in donor substrate binding (Fig 3c). Mapping, however, points to a much larger active site pocket, in which acceptor substrate binding may be stabilized by the following conserved residues: E243, Q260, S443, V446, Q448, and M464 (Fig 3c). Sequence conservation within this region of Och1 especially, across multiple yeast and mold species, reinforces the functional importance of these residues in the Och1 active site, such that it is likely the residues present have been evolutionarily determined to be necessary for substrate specificity. This evidence of evolutionary conservation may also be applied to the biological role of Och1 in initiating fungal cell wall biosynthesis, in support of the literature evidence, in that nature appears to have identified and conserved key residues to facilitate α-1,6-mannosylation of the eukaryotic *N*-GlcNAc_2_Man_8_ glycan [4,10,13,14].

### Structural homology search reveals Och1 shares limited similarity to other GT-A folds

A Dali Server full PDB search indicates Och1 to be structurally unique; as no proteins with a z-score greater than 15 were found (Table 2) [39]. Not too surprisingly, the two enzymes that are most similar to Och1 are also the only other available GT family 32 proteins present in the PDB, PaToxD (Jank et al, 2013. PDB: 4MIX) and YeGT (Schneider et al, 2024. PDB: 8OVT) [43,44]. Both PaToxD and YeGT are insect bacterial toxins which perform Rho protein modifications by tyrosine GlcNAcylation [43,44]. Neither protein share functional or substantial structural similarity to Och1. Yet, despite originating from differing domains of life and performing different glycosylations, YeGT is still the top match to Och1, with a z-score result of 14.4 (Table 2). Furthermore, Och1 appears to be remarkably structurally unique in relation to other known mannosyltransferases, the best of which being Mnt2 an *S. cerevisiae* α-1,3-MT, sharing just 11% identity, with a z-score of 8.5 (Hira et al., 2023 PDB: 7XJV) (Table 2) [45]. Superposition of Och1 to full sequence length PaToxD, YeGT, and Mnt2 yields little agreement, with an RMSD of 9.073 Å, 6.034 Å, and 24.382 Å, respectively (in contrast, PaToxD and YeGT superimposition calculates an RMSD of 0.885 Å) (S4 Fig).

**Table 2:** Summary of relevant Dali search results compared to Och1 structure.

Protein	PDB	GT	Transferase Class	RMSD (Å):	Lali^i^	Nres^ii^	%ID^iii^	z-score
YeGT	8OVT	32	GlcNAc-^v^	3.0	191	288	18	**14.4**
PaToxG	4MIX	32	GlcNAc-	3.0	186	277	19	**14.2**
TpeL	9BON	44	Glucosyl-	3.4	200	548	14	**14.1**
TcdB	6OQ7	44	Glucosyl-	3.2	191	541	14	**13.9**
YGT	6RTH	--- ^iv^	Glucosyl-	2.9	191	496	15	**13.8**
TcdA	6RTH	44	Glucosyl-	3.0	189	542	16	**13.4**
A064R	2P6W	---	Rhamnosyl-	3.3	167	206	11	**10.2**
Lgt1	2WZF	88	Glucosyl-	5.3	193	515	10	**10.1**
SseK2	5H62	---	GlcNAc-	3.3	179	301	14	**9.8**
Large1	7ZVJ	8, 49	Xylosyl-, glucuronyl-	3.6	179	583	15	**9.1**
Mnt2	7XJV	71	Mannosyl-	3.9	203	507	11	**8.5**
Xxylt1	4WLG	8	Xylosyl-	3.9	182	290	11	**8.4**
Xxt1	6BSU	34	Xylosyl-	4.1	183	337	10	**8.1**
Apre_0416	3TZT	8	Unknown	3.4	149	234	13	**7.5**
LgtC	1GA8	8	Galactosyl-	4.0	174	278	10	**7.5**
GlyE	5GVV	8	Galactosyl-	3.6	157	392	12	**6.8**
Kre2/Mnt1	1S4N	15	Mannosyl-	3.6	179	337	9	**6.7**
Ktr4	5A07	15	Mannosyl-	4.1	167	395	7	**5.9**
Mgat1	1FOA	13	GlcNAc-	3.6	140	342	10	**5.2**
GalNac-T7	6IWQ	27	GlcNAc-	3.7	150	546	11	**4.9**
GaNTase-T1	1XHB	27	GlcNAc-	3.6	143	447	10	**4.3**
GlfT2	4FIX	2	Gal*f*-^vi^	4.0	145	629	14	**4.2**
Mnn9	3ZF8	62	Mannosyl-	4.2	133	288	10	**4.2**
FbiD	6BWH	---	Guanylyl-	3.8	120	207	10	**3.3**
Chs1	7XS7	2	Chitin synthase	4.1	148	729	12	**2.9**

^i^Lali = number of aligned C-alpha atoms.

^ii^Nres = number of residues in the target structure.

^iii^%ID = percent identity of aligned amino acids.

^iv^--- = non-classified.

^v^*N*-acetylglucosaminyl-.

^vi^Galactofuranosyl-.

Structure characterization of Och1 reveals the protein somewhat strays from the typical GT-A folding pattern [25,46]. A conventional GT-A fold single domain consists of a “bed-like” core of approximately seven β-strands surrounded by a varying number of ⍺-helices forming an ⍺/β/⍺ sandwich. Between the β4 and β5 strands, normally exists an antiparallel β4’ strand which is immediately preceded by a short loop housing the DxD motif. The β4’ forms a small antiparallel sheet with a second β7’ strands [25,46]. In contrast, Och1 possesses its DxD motif flanked the β3 and β3’ strands. Where it is the β3’ strand that forms the GT-A signature small antiparallel sheet with a β7’ exterior to the ⍺/β/⍺ sandwich (Fig 2a and 2b) [25,46]. Nevertheless, general alignment of the β-sheet core is possible, which enables us to envision the nucleotide sugar binding pocket location on Och1, based on PaToxD’s binding to UDP-GlcNAc and known GT-A common core elements (S4 Fig).

### Och1-substrate complex molecular modeling is consistent with known Och1 mannosyltransferase activity

**Fig 4:**
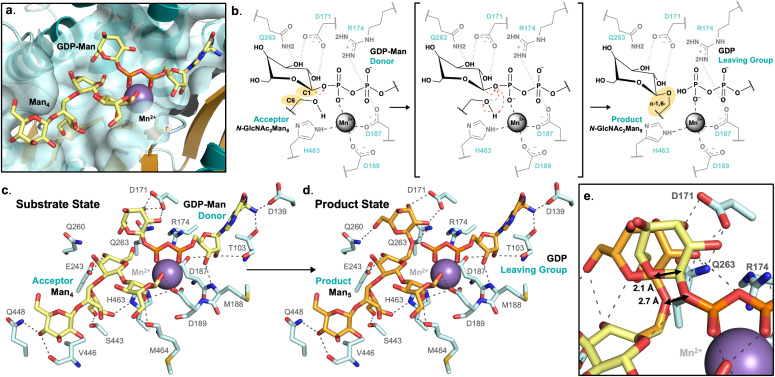
Molecular modeling of nucleotide-sugar donor and acceptor substrates to Och1 crystal structure. **a.** Och1 active site pocket modeled in the substrate bound state, with Mn^2+^ metal cation, the GDP-Man donor, and Man_4_ glycan D1 branch acceptor substrate. **b.** Schematic representation of the S_N_i-like mechanism of action Och1 is thought to employ. **c.** Modeled substrate (GDP-Man donor and Man_4_ acceptor) bound state shown to be stabilized by Och1 active site residues, and the subsequent **d.** product (GDP leaving group and Man_5_ glycan branch) bound state. **e.** Overlayed modeling of the substrate and product bound states, displaying the donor mannose residue pivot about the anomeric C1 carbon.

*In silico* placement of substrates bound to *Sc*Och1 highlights the real size of the glycosyltransferase ligand binding pocket, measuring approximately 30 Å across and with solvent accessible surface area of ~460 Å^2^ (as measured for residue atoms within 4.0 Å of modeled acceptor substrate) (Figs 3, 4 and S4). Within the donor binding site, conserved residues at T103 and D139 likely stabilize guanosine binding, at the β1 strand C-terminus (Fig 4c and 4d). Whereas the GDP-mannose moiety is poised to interact via the C2 and C3 hydroxyl groups, forming a hydrogen-bonding network with the conserved residues Q263, D171, R174, and D187 (of the DxD motif) – occurring on the back-face of GDP-Man (Fig 4c and 4d). Placement of the donor C2 and C3 hydroxyls in close proximity to Q263 and D171 allows for the necessary orientation of the anomeric carbon such that an α-1,6-linkage with the accepting mannose is possible (Fig 4d). The accepting mannose residue appears to be stabilized by possible C4 hydroxyl group hydrogen bond with a conserved E243 pocket residue, making the C6 acceptor hydroxyl group available to the donor anomeric carbon (Fig 4c and 4d). Modeled transfer of the donor mannose residue onto the acceptor would result in a slight pivot, such that the anomeric C1 carbon would travel a calculated distance of ~2.1 Å, thus continuing to allow for donor C2 and C3 hydroxyl interactions with Q263 and D171, as well as forming another hydrogen bond between the donor’s C6 hydroxyl and Q260 (Fig 4d).

Och1 functions as the initiator of mannan synthesis by the addition of a fungi specific α-1,6-Man onto the universal eukaryotic *N*-GlcNAc_2_Man_8_ glycan, occurring along the glycan’s D1 branch (Fig 1) [13,17]. Specifically, the accepting mannose moiety within the larger glycan is also engaged in two α-1,2- and α-1,3-glycosidic linkages with its neighbouring mannose subunits though its C1 and C2 hydroxyl groups (Fig 1) [17]. Confidence in this placement of donor and acceptor mannose residues, forming an α-1,6-linkage, is also supported by the resulting orientation where the acceptor mannose’s C1 and C2 hydroxyl groups can form further α-1,2- and α-1,3-linkages in the same pocket (Fig 4). This matches the known Och1 *N*-glycan acceptor substrate glycosylation pattern, allowing for positioning of all four D1 branch mannose residues. Wherein, extended modeling of each mannose residue indicates substrate stabilization via hydrogen-bonding with Och1 pocket residues E243, Q260, S443, V446 (main chain), Q448, and M464 (main chain) (Fig 4c and 4d).

Fifty-two residues line the pocket of the *Sc*Och1 active site; of which, thirty-one are situated near the donor, and twenty-three near the acceptor site. Of these, thirty-two are either nearly or completely conserved across at least twenty-two yeast and mold Och1 homologs (Fig 3). Fig 3b and 3d shows modeling of a truncated Man_4_ glycan (the D1 Man(α1–2)Man(α1–2)Man(α1–3)Man(β1–4) branch) within the Och1 active site, where a number of conserved residues may be involved in a binding orientation that would allow for Och1 substrate specificity for the eukaryotic *N*-GlcNAc_2_Man_8_ core D1 branch (Fig 3). Confidence in the biological relevance of our substrate modeling is not only boosted by the highly conserved nature of the Och1 active site and architecture uniquely suited to accommodate the specific *N*-GlcNAc_2_Man_8_ glycosidic linkage patterning, it also doubles to increase confidence in Och1 substrate binding specificity as is reported in the literature [4,17].

## Discussion

### Molecular modeling supports prediction of front-faced S_N_i-like retaining mechanism for Och1

An evolutionary analysis of GT-A folds by Taujale et. al. has identified several features that are often present, despite varying catalytic architectures amongst these enzymes [42]. Notably, the DxD motif is nearly always observed, and may often be accompanied by an xED motif. When simultaneously present, the first aspartic acid of the DxD motif is involved in metal coordination, while the aspartic acid residue in the xED motif acts the nucleophile that donates the electron required for transfer reaction. As mentioned above, our structure shows that in *Sc*Och1, the DxD motif is indeed located at the expected GT-A folded position (Fig 2). However, the xED motif is not found. The absence of the xED motif, necessarily, impacts the details of the mechanism by which Och1 may transfer the sugar moiety from GDP-Man to the universal *N*-GlcNAc_2_Man_8_ glycan. One possible mechanism put forward in the literature for ret-GTs entails an unconventional front-facing S_N_i (substitution nucleophilic internal) reaction [47,48]. This has been proposed for ret-GTs lacking any apparent beta-face positioned nucleophilic activating motif. Wherein, the decomposing leaving group alternatively provides the necessary nucleophile, as an ion pair, and is held on the same face as the leaving group [47,48].

Our molecular modeling of the nucleotide-sugar donor, glycan acceptor, and reaction products provide potential before-and-after snapshots of Och1 activity. Confidence in Fig 4 modeling is supported by high conservation of the Och1 active site pocket (Fig 3). Donor and acceptor modeled placement, shown in Fig 4, places both the donor’s anomeric carbon C1 and the acceptor’s C6 hydroxyl group within 2.7 Å (Fig 4e). The accepting C6 hydroxyl is within reaction distance with what would be the newly cleaved GDP leaving group phosphate such that the necessary theoretical oxocarbenium ion transition state may form (Fig 4) [47,48]. This would be in line with the proposed S_N_i retaining mechanism, allowing for a glycosidic transfer while maintaining a net retention of the anomeric carbon’s stereochemistry (Fig 4b). A similar conclusion was also made with the retaining xylosyltransferase, Xxylt1, in which an appropriately placed activating nucleophilic residue (i.e., the xED motif) is also absent (PDB: 4WLG) (Table 2, S4 Fig) [49]. Molecular modeling may fail to definitively determine the exact mechanism of transfer. However, data presented here, in combination with considerable evolutionary conservation across Och1 homologs, does continue to correspond with the literature and prevailing theories of S_N_i retaining glycosyltransferase mechanisms of action [25,47,48].

### Highly conserved Och1 active site architecture necessitates *N*-GlcNAc_2_Man_8_ branch binding specificity

Prior functional studies have long since confirmed the biochemical role of Och1 in initiating fungal outer cell wall mannan synthesis. Nuclear magnetic resonance (NMR) and fast atom bombardment mass spectrometry (FAB-MS) work have shown the specific α-1,6-linked mannose product as a result of Och1 *in vitro* activity [15,16]. However, in the absence of structural data, the strategy by which Och1 does this has remained unknown.

The highly conserved and specific structure of the *Sc*Och1 active site appears to demand a specific eukaryotic *N*-GlcNAc_2_Man_8_ core binding orientation (Figs 3 and 4). The *N*-GlcNAc_2_Man_8_ glycan core possesses two major high-mannose branches, with differing glycosylation patterns; branches D1 and D3 [13]. The glycan, being central to eukaryotic biology, is bound and/or modified by many different glycosyltransferases in addition to Och1, depending on the species, tissue, cellular localization, and/or pathway at play. Reminiscent of a lock-n-key, our modeling efforts show the ~ 460 Å^2^ conserved pocket is constructed such that only the α-1,2- and α-1,3-linked mannose branch of the larger *N*-glycan (the D1 Man(α1−2)Man(α1−2)Man(α1−3)Man(β1−4) branch) may fit (Figs 3 and 4d and 4f). Not only would the glycosidic patterning of the α-1,6-linked mannose branch be unable to fit within the Och1 active site, as our structure makes clear, the addition of another α-1,6-Man is not chemically possible on the D3 branch; as each monomer is engaged in a prior α-1,6-linkage (Fig 4f). This can further rationalize the glycosidic modification specifically to the (α-1,2)Man(α-1,3) residue of the D1 branch due to the positioning of each D1 branch residue, particularly given the terminal binding to the Man(D1) residue towards the exterior of the pocket (Figs 3 and 4). With a conserved active site so perfectly designed to house such a large and specific portion of the *N*-glycan branch, our model can be assumed with confidence.

### Molecular modeling of Och1 substrate-bound complex is consistent with other known high-mannose bound glycosyltransferases

The extended nature of the donor binding pocket in Och1 is unusual. A PDB search reveals a total of three known glycosyltransferases in complex with a high-mannose glycan acceptor group possessing three or more sugar subunits. These are: human β-1,4-galactosyltransferase-1 (β4Gal-T1) (PDB: 2AE7, 2AEC, 2AES, 2AGD, 2AH9), human α-1,6-fucosyltransferase (Fut8) (PDB: 6X5R, 6X5S, 6X5U), and human α-mannoside β-1,6-*N*-acetylglucosaminyltransferase V (MGat5) (PDB: 5ZIC, 6YJV, 6YJU 6YJS) [50–53]. Each are human GTs belonging to differing CAZy families and folds, and each performs a different glycosyl transfer reaction (Table 3). Multiple structures of β4Gal-T1 bound to varying trisaccharide glycans are available for analysis, each of which shows the protein only binds to terminal glycan monomer GlcNAc(β1,2) residue [50] (Table 3). As such, the β4Gal-T1 presents a binding site with specificity for the glycan’s terminal glycan, where β4Gal-T1 forms a Gal(β1,4)GlcNAc linkage [50]. Alternatively, Fut8 and MGat5 both perform glycosyl transfer reactions upon an internally linked monomer within a much larger glycan [51,53]. This is conceptually similar to acceptor binding for Och1. Fut8 (PDB: 6X5R) in complex with GlcNAc_4_Man_3_ shows hydrogen bonding interactions with six of the seven sugar subunits [49] (Table 3). While MGat5 (PBD: 6YJS), in complex with GlcNAc_1_Man_3_, forms direct hydrogen bonding with three of the five monomers [53] (Table 3). Fut8 and MGat5 both present binding pockets that allow, not only large glycan binding, but for strategic binding that permits glycosylation of an internally glycosidically linked sugar residue. Our molecular modeling of substrate in complex with Och1 is consistent with this (Table 3, S5 Fig). Moreover, accounting for acceptor site surface area and number of hydrogen bonds per interacting sugar monomers (those that are directly bound by the enzyme), molecular modeling of substrate binding to Och1 also approximately scales with the number of interactions formed by both MGat5 and Fut8 (Table 3, S5 and S6 Figs).

**Table 3:** Och1 compared to PDB glycosyltransferases in complex with high-mannose substrate.

Protein	PDB	Species	Class	GT-Family			Acceptor Site Surface (Å^2^)
Och1	9N3S	Yeast	Mannosyl-	32	GT-A	Retaining	460
MGat5	6YJS	Human	Acetylglucosaminyl-^i^	18	---	Inverting	500
Fut8	6X5R	Human	Fucosyl-^ii^	23	GT-B	Inverting	770
β4Gal-T1	2AEC	Human	Galactosyl-^iii^	7	GT-A	Inverting	400
**Protein**	**Glycan Subunit** ^iv^			**Atom Contacts**			**Distance (Å)**
Och1	Man(α1,2)			HE21_*Q448*_ --- O4_*Man*_			2.1
				O_*V446*_ --- OH4_*Man*_			2.3
	Man(α1,2)			OG_*S443*_ --- OH6_*Man*_			1.9
				HD1_*H463*_ --- O4_*Man*_			2.2
	Man(α1,3)^*^			OE2_*E243*_ --- OH4_*Man*_			2.1
	Man(β1,4)			O_*M464*_ --- OH2_*Man*_			2.2
				O_*M464*_ --- OH6_*Man*_			2.1
MGat5	GlcNAc(β1,2)			OE2_*E297*_ --- HN2_*GlcNAc*_			1.9
				OD1_*D365*_ --- OH4 _*GlcNAc*_			1.8
				H_*S366*_ --- O3_*GlcNAc*_			2.1
				HZ1_*K541*_ --- O6_*GlcNAc*_			2.2
				HZ3_*K541*_ --- O_*GlcNAc*_			3.0
	Man(α1,6)^*^			HZ2_*K541*_ --- O3_*Man*_			2.0
	GlcNAc(β1,2)			HE22_*Q345*_ --- O6_*GlcNAc*_			2.9
Fut8	GlcNAc(β1,2)			H06_*H535*_ --- O6_*GlcNAc*_			1.7
				OD2_*D495*_ --- H02_*GlcNAc*_			2.0
	GlcNAc(β1,2)			H03_*EQ503*_ --- O7_*GlcNAc*_			2.6
				H03_*G501*_ --- O7_*GlcNAc*_			2.0
	Man(α1,6)			O_*V531*_ --- OH4_*Man*_			2.8
				H08_*Q502*_ --- O_*Man*_			2.6
	Man(β1,4)			OD1D_*495*_---OH4_*Man*_			2.2
	GlcNAc(β1,4)			OE1_E373_ --- OH6_GlcNAc_			2.2
				H07_*Q470*_ --- O7_*GlcNAc*_			2.2
	GlcNAc(β1-^*^			OD2_*D295*_ --- OH3_*GlcNAc*_			2.2
				H07_*Q470*_ --- O4_*GlcNAc*_			2.9
				OE2_*E373*_ --- O6 _*GlcNAc*_			2.0
β4GalT1	GlcNAc(β1,2)^*^			Ho1_*G312*_ --- O3_*GlcNAc*_			1.9
				OF2_*D315*_ --- H02_*GlcNAc*_			1.8
				H09_*R355*_ --- O7_*GlcNAc*_			1.7

^i^*N*-acetylglucosaminyltransferase.

^ii^Fucosyltransferase.

^iii^Galactosyltransferase.

^iv^See S6 Fig for schematic representation of acceptor substrate.

^*^Acceptor moiety for modification.

While it is recognised that structural data remains limited, analysis of this literature indicates an emerging trend for glycosyltransferases tasked with modifying sites within an internally linked glycan. Suggesting, internally linked glycan modifying GT binding pockets must be large enough to accommodate large portions of the target glycan and with ample specificity for multiple saccharide residues. Therein access to the acceptor moiety’s target site may be granted such that it faces towards the interface of the nucleotide donor binding pocket, with optimal orientation for the desired glycosyltransfer to take place. More structural data concerning these types of glycosyltransferases is needed to confirm this proposed hypothesis.

## Conclusion

Here, the first X-ray crystal structure of *Saccharomyces cerevisiae* Och1 has been solved to a resolution of 2.0 Å, R/R_free_: 0.1978/0.2428 (Fig 2). This structure confirms an evolutionarily conserved yet structurally unique GT-A folding pattern that has extensive *N*-glycosylation present at positions N203 and N281. In the absence of a suitable β-face catalytic residue in the Och1 active site, Och1 is proposed to perform a front-face S_N_i reaction [47]. This, in combination with our substrate molecular modeling to generate predicted Och1 substrate-bound and product-bound complexes, indicates Och1 has the likely capacity to bind a large high-mannose glycan, with target specificity for the modification of an internally linked mannose residue. In light of literature evidence indicating deletion of Och1 results in diminished cell growth and virulence, Och1 has long been considered an attractive option for pharmacological study [10–12]. Our work serves to further advance Och1 as a potential fungi specific drug target for cell wall synthesis inhibition. The highly conserved, and large acceptor substrate binding pocket with a predicted unique binding specificity for a large internally linked glycan substrate, may enable structure guided design towards a high-mannose acceptor mimetic. Though it is recognized that target engagement can be challenging, such an inhibitor provides an avenue for pursuing the development of novel antimycotic treatments.

## Supporting information

S1 Fig Comparison between Och1 predicted by AlphaFold and the X-ray crystal structure of Och1. Areas of low confidence in the AlphaFold model are shown in orange, which correspond with areas of missing electron density in the crystal structure.
(TIF)

S2 Fig Calculated root mean square difference (RMSD) of backbone and sidechain atoms, plotted by each Och1 amino acid residue. RMSD (Å) values are shown comparing atoms belonging to **a.** Och1 crystal chains A and B, **b.** AlphaFold predicted Och1 model and Och1 Chain A, and **c.** AlphaFold predicted Och1 model and Och1 Chain B.
(TIF)

S3 Fig Molecular modeling of nucleotide-sugar donor and acceptor substrates to Och1 crystal structure. Och1 shown by surface rendering with each modeled ligand bound to the glycosyltransferase active site. Molecular modeling showing sequentially **a.** AlphaFold3 predicted GDP and Mn^2+^ binding, followed by MOE modeled binding of **b.** GDP-Man and acceptor mannose moiety, **c.** GDP and α-1,6-mannobiose reaction product, **d.** GDP-Man and Man_4_ representation of universal *N*-linked eukaryotic glycan, and **e.** GDP and Man_5_ reaction product state.
(TIF)

S4 Fig Superimposition of Och1 compared to Dali search results. Showing **a-d.** PaToxG (PDB: 4MIX), YeGT (PDB: 8OVT), **e-f.** α-1,3-mannosyltransferase Mnt2 (PDB: 7XJV), and **g-h.** α-1,3-xylosyltransferase Xxylt1 (PDB: 4WLG).
(TIF)

S5 Fig Och1 binding pocket surface area compared to PDB glycosyltransferases in complex with high-mannose substrate. **a.** Solvent accessible surface area (Å^2^) measured to 4 Å from substrate bound for all glycosyltransferase substrate acceptor site atoms (dark blue). Total number of hydrogen bonds formed by each protein is shown in light blue. Number of glycan moieties directly bound by enzyme indicated in orange. **b.** Per number of directly bound glycan moieties: averaged acceptor surface area (Å^2^) (dark blue) and average number of hydrogen bonds formed (light blue).
(TIF)

S6 Fig Acceptor glycans bound to known high-mannose bound glycosyltransferase (GT) PDB structures. Schematic representation of the acceptor glycan portion included in high-mannose bound to GT PDB structures compared to Och1 molecular modeled substrate-bound state. Glycan moieties directly bound by enzyme are indicated with a yellow background. Acceptor moiety to be modified by the GT are denoted by * symbol. **a.** Och1 (PDB: 9N3S) acceptor substrate Man_4_. **b.** β4GalT1 (PDB: 2AEC) acceptor substrate GlcNAc_1_Man_2_. **c.** Fut8 (PDB: 6X5R) acceptor substrate GlcNAc_4_Man_3_. **d.** MGat5 (PDB: 6YJS) acceptor substrate GlcNAc_2_Man_3_.
(TIF)

S1 Table *N*-glycan glycosyltransferases involved in early fungal cell wall synthesis. (DOCX)

S1 Data Och1 substrate bound sate. Coordinate file in PDB format, containing a 3D model of Och1 in the substrate bound state. File contains Och1 bound to GDP-mannose and Man_4_ acceptor glycan.
(PDB)

S2 Data Och1 product bound sate. Coordinate file in PBD format, containing a 3D model of Och1 in the product bound state. File contains Och1 bound to GDP leaving group and Man_5_ product.
(PDB)
