# Supplementary figures and images for: Crystal structure of the fungal mannosyltransferase Och1 reveals active site primed for N-glycan binding
Source: PLoS One. 2025 Jul 31;20(7):e0329259. doi: 10.1371/journal.pone.0329259 (PMC12312940; doi:10.1371/journal.pone.0329259)

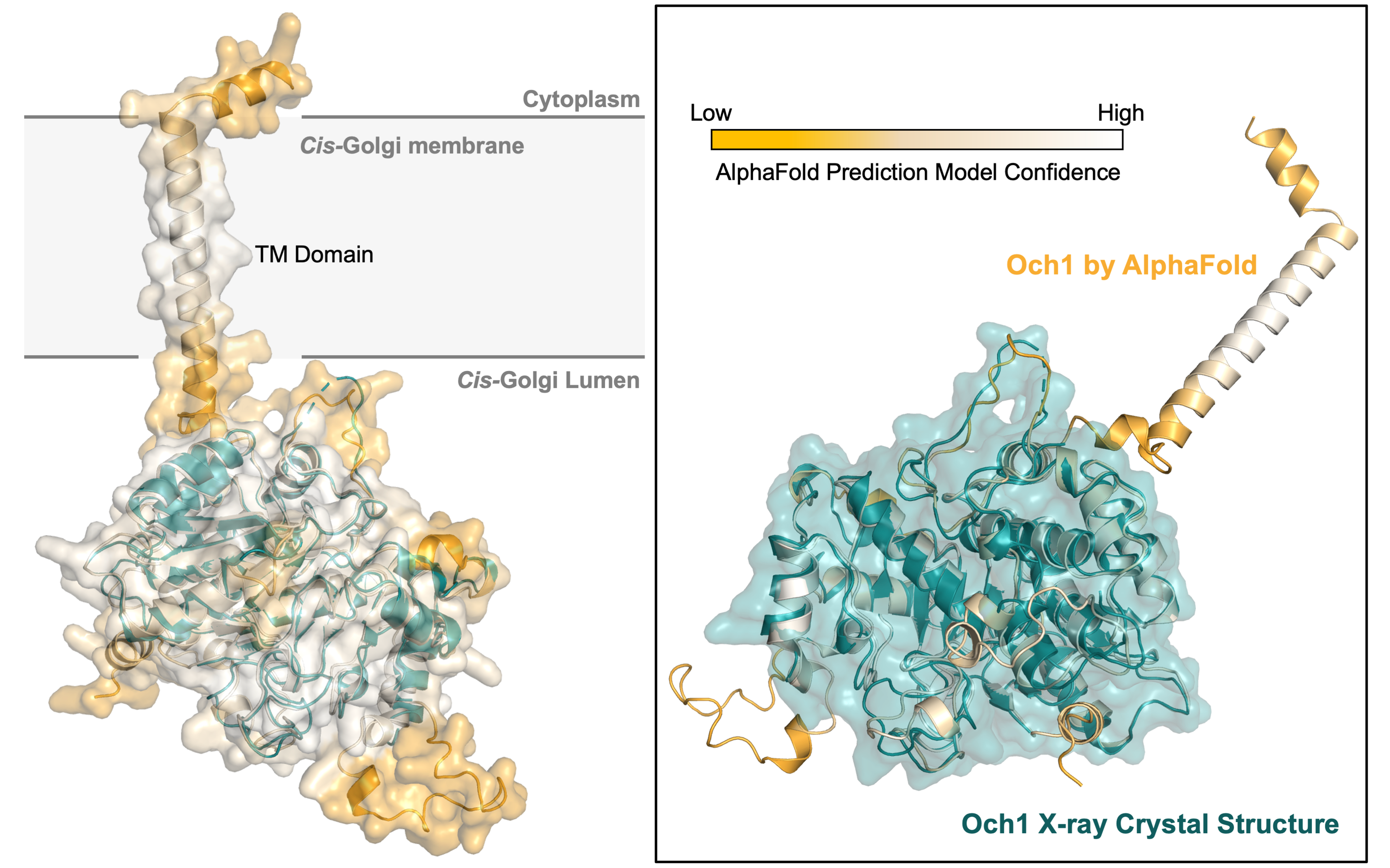

Supplement: S1 Fig — Areas of low confidence in the AlphaFold model are shown in orange, which correspond with areas of missing electron density in the crystal structure. (TIF) [file pone.0329259.s001.tif]

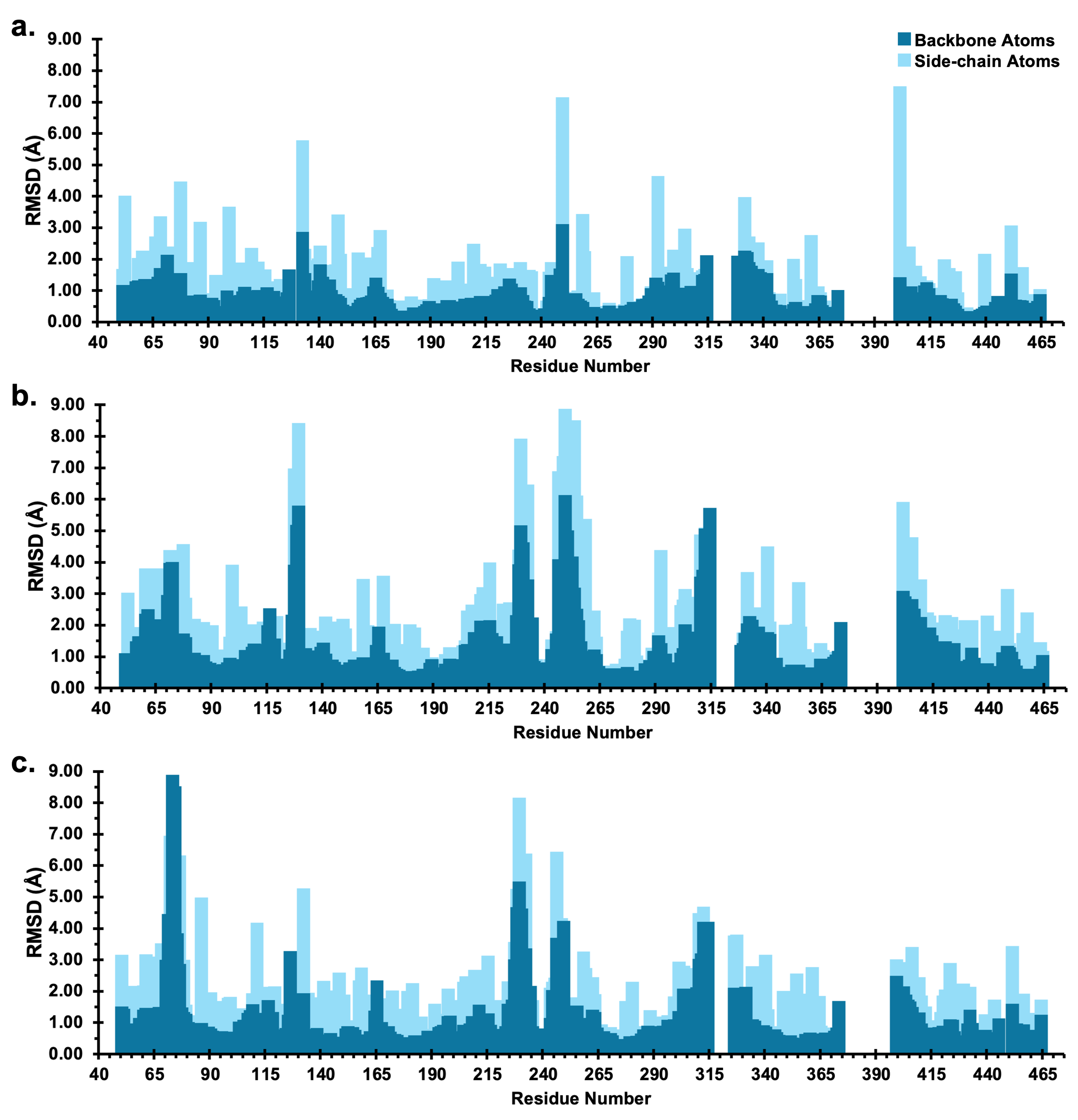

Supplement: S2 Fig — RMSD (Å) values are shown comparing atoms belonging to a. Och1 crystal chains A and B, b. AlphaFold predicted Och1 model and Och1 Chain A, and c. AlphaFold predicted Och1 model and Och1 Chain B. (TIF) [file pone.0329259.s002.tif]

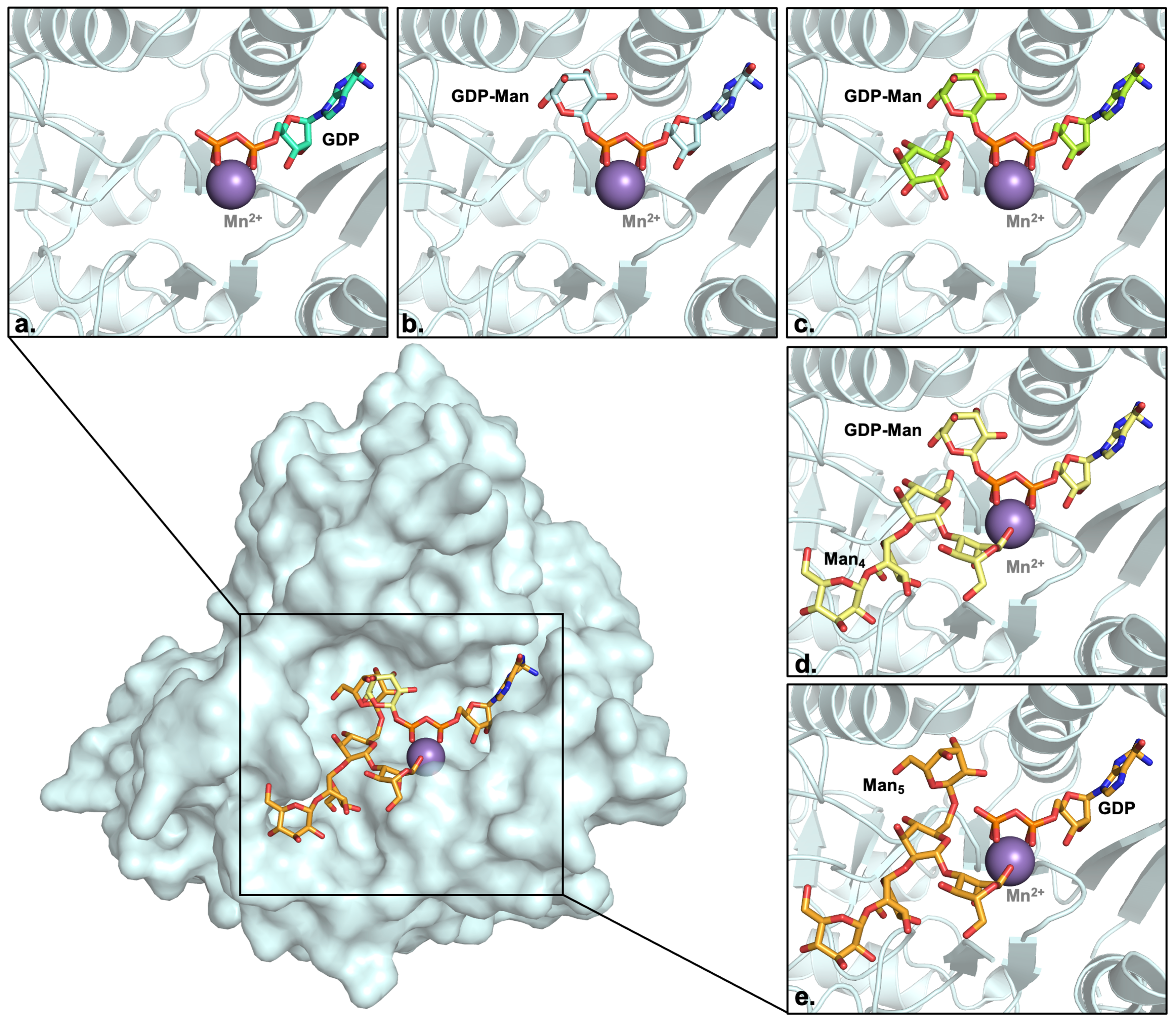

Supplement: S3 Fig — Och1 shown by surface rendering with each modeled ligand bound to the glycosyltransferase active site. Molecular modeling showing sequentially a. AlphaFold3 predicted GDP and Mn2+ binding, followed by MOE modeled binding of b. GDP-Man and acceptor mannose moiety, c. GDP and α-1,6-mannobiose reaction product, d. GDP-Man and Man4 representation of universal N-linked eukaryotic glycan, and e. GDP and Man5 reaction product state. (TIF) [file pone.0329259.s003.tif]

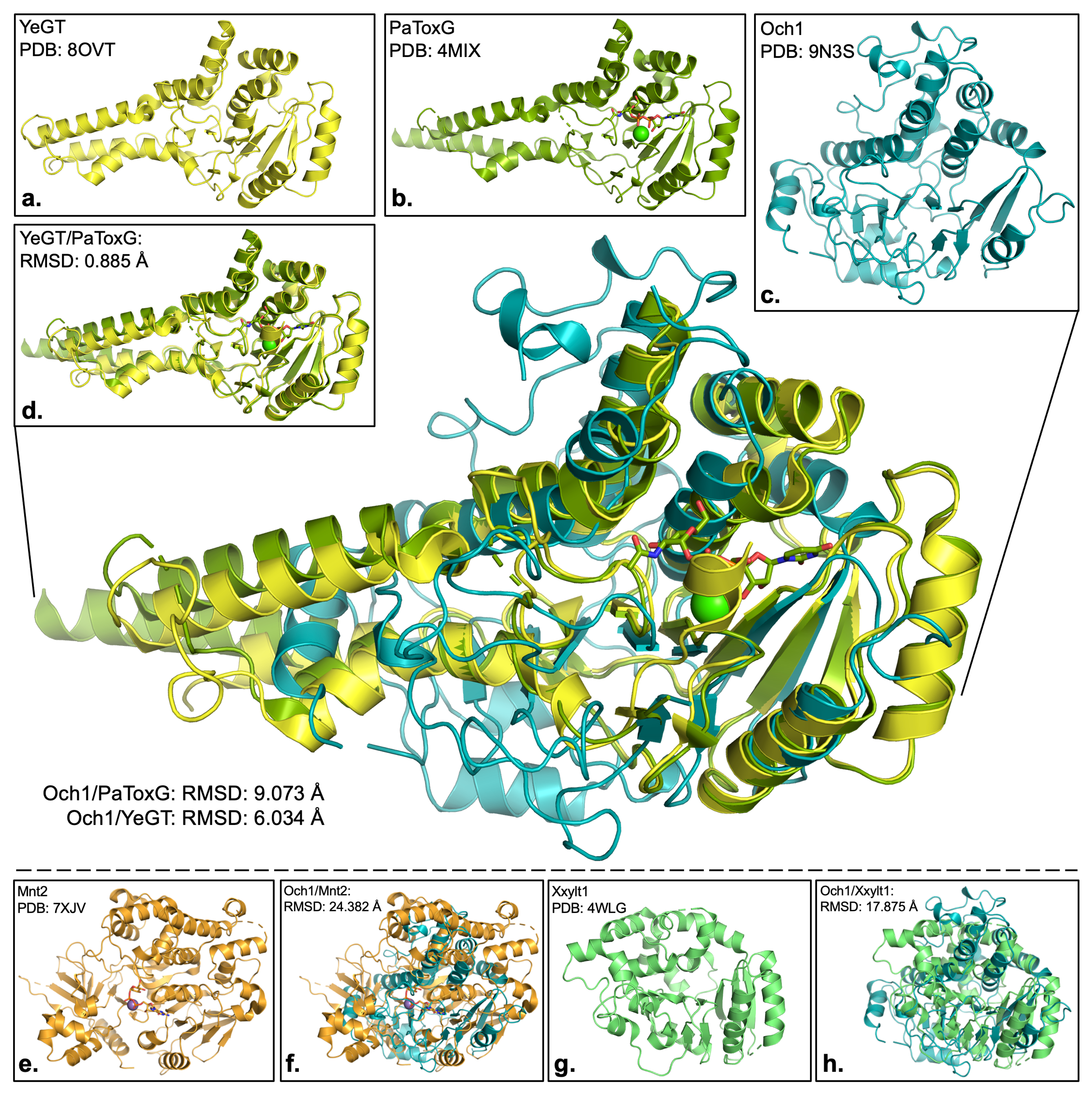

Supplement: S4 Fig — Showing a-d. PaToxG (PDB: 4MIX), YeGT (PDB: 8OVT), e-f. α-1,3-mannosyltransferase Mnt2 (PDB: 7XJV), and g-h. α-1,3-xylosyltransferase Xxylt1 (PDB: 4WLG). (TIF) [file pone.0329259.s004.tif]

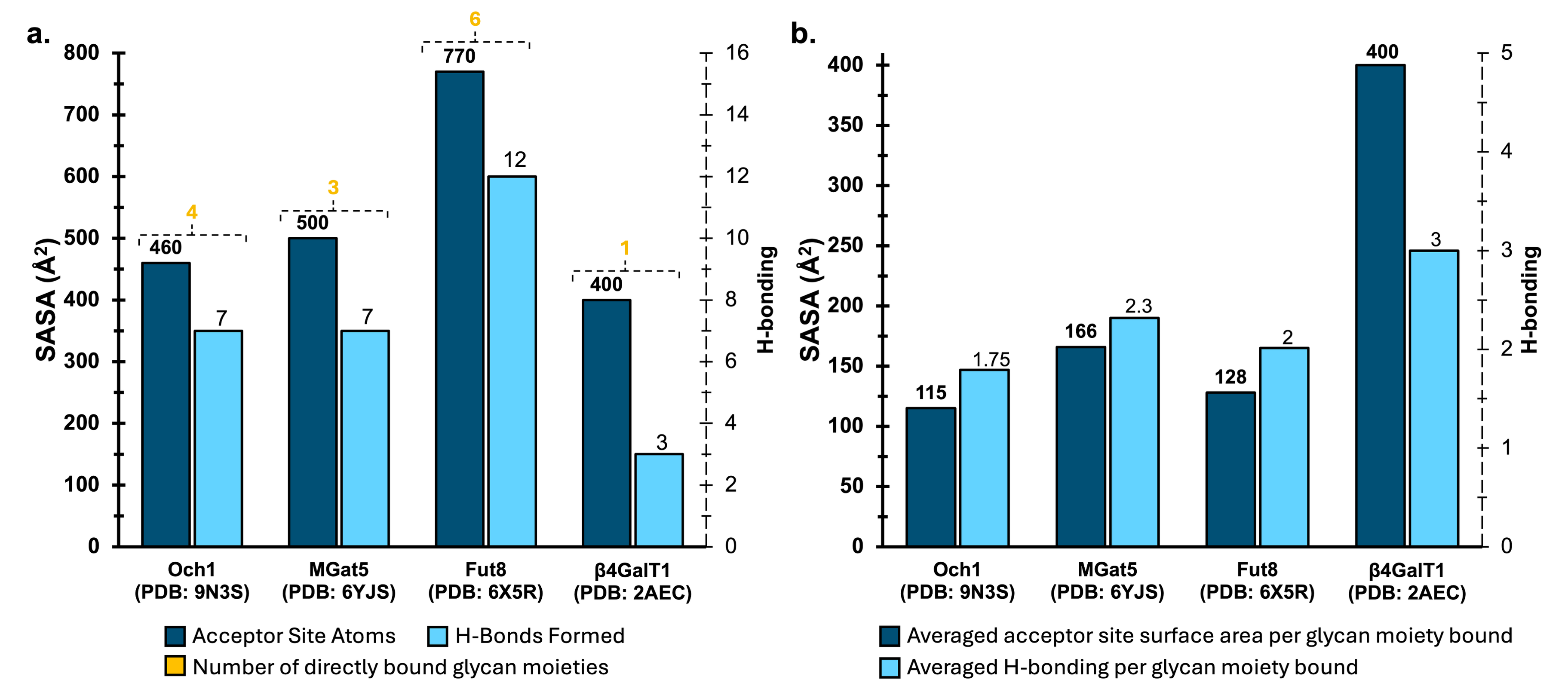

Supplement: S5 Fig — a. Solvent accessible surface area (Å2) measured to 4 Å from substrate bound for all glycosyltransferase substrate acceptor site atoms (dark blue). Total number of hydrogen bonds formed by each protein is shown in light blue. Number of glycan moieties directly bound by enzyme indicated in orange. b. Per number of directly bound glycan moieties: averaged acceptor surface area (Å2) (dark blue) and average number of hydrogen bonds formed (light blue). (TIF) [file pone.0329259.s005.tif]

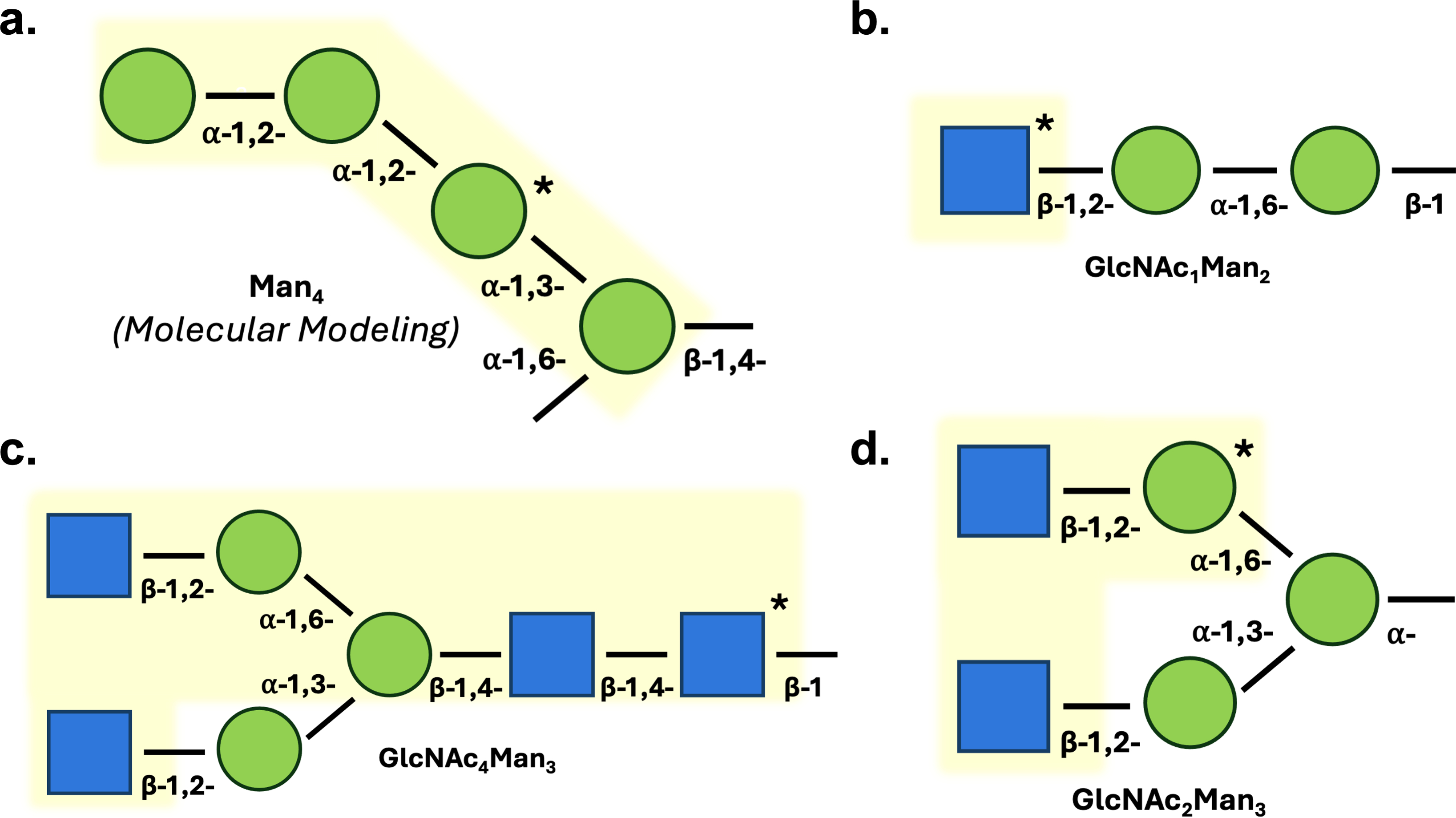

Supplement: S6 Fig — Schematic representation of the acceptor glycan portion included in high-mannose bound to GT PDB structures compared to Och1 molecular modeled substrate-bound state. Glycan moieties directly bound by enzyme are indicated with a yellow background. Acceptor moiety to be modified by the GT are denoted by * symbol. a. Och1 (PDB: 9N3S) acceptor substrate Man4. b. β4GalT1 (PDB: 2AEC) acceptor substrate GlcNAc1Man2. c. Fut8 (PDB: 6X5R) acceptor substrate GlcNAc4Man3. d. MGat5 (PDB: 6YJS) acceptor substrate GlcNAc2Man3. (TIF) [file pone.0329259.s006.tif]
