# Supplementary material for: Crystal structure of the fungal mannosyltransferase Och1 reveals active site primed for N-glycan binding
Source: PLoS One. 2025 Jul 31;20(7):e0329259. doi: 10.1371/journal.pone.0329259 (PMC12312940; doi:10.1371/journal.pone.0329259)
Supplement: S1 Table — (DOCX) [file pone.0329259.s007.docx]

**S1 Table. *N*-glycan glycosyltransferases involved in early fungal cell wall synthesis**

|  | Protein/Subunit | GT Family | GT Fold | GT Mechanism |
| --- | --- | --- | --- | --- |
|  | Och1 | 32 | GT-A | Retaining |
| M-Pol 1 | Van1 | 62 | GT-A | Retaining |
|  | Mnn9^i^ | 62 |  |  |
| M-Pol II | Anp1 | 32 |  |  |
|  | Mnn10 | 34 |  |  |
|  | Mnn11 | 34 |  |  |
|  | Hoc1^ii^ | 32 | GT-A | Retaining |

^i^Mnn9 crystal structure PDB: 3ZF8 (14).

^ii^Hoc1 as a fifth M-Pol II subunit is debated.
